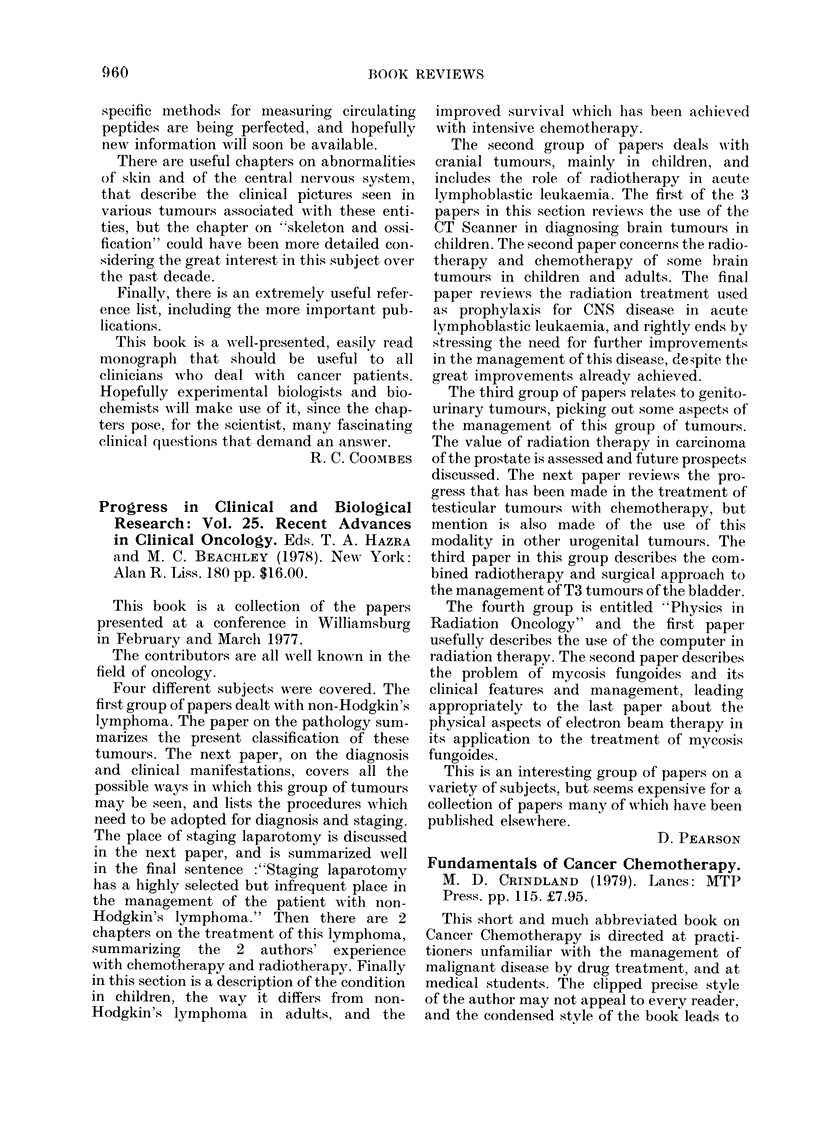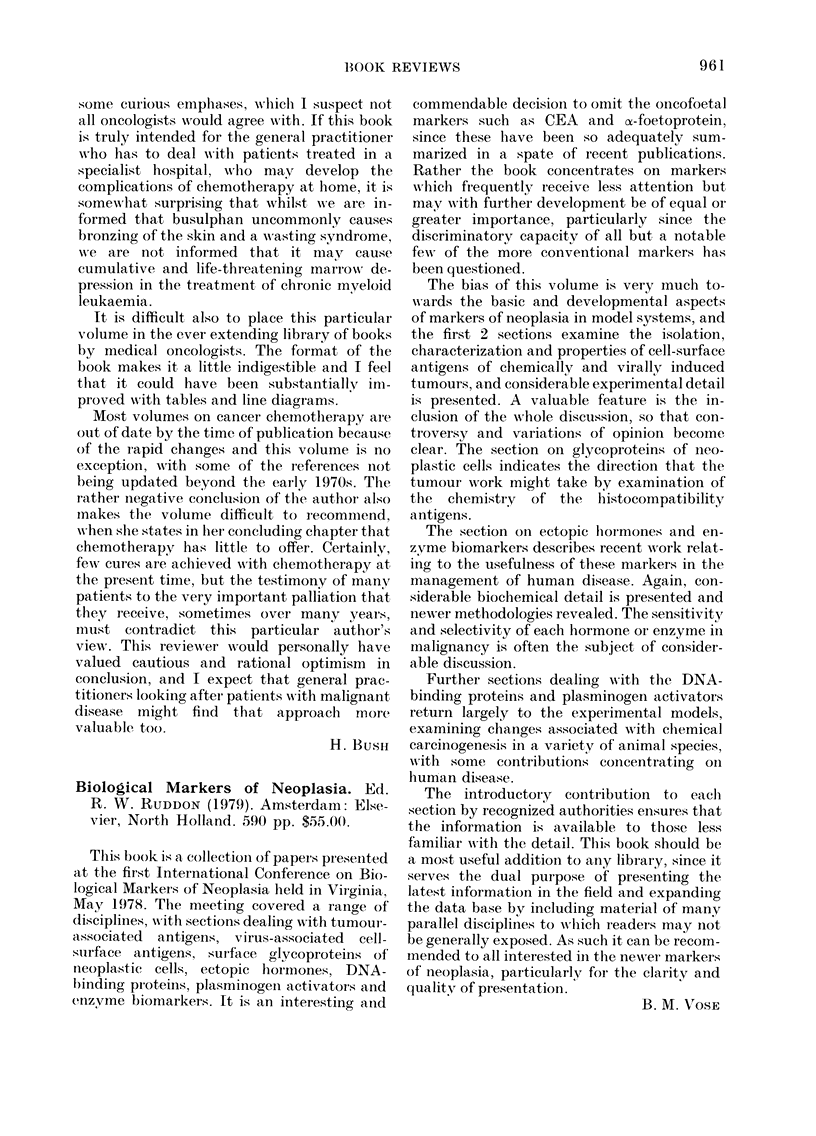# Fundamentals of Cancer Chemotherapy

**Published:** 1979-12

**Authors:** H. Bush


**Fundamentals of Cancer Chemotherapy.** M. D. CRINDLAND (1979). Lancs: MTP Press. pp. 115. £7.95.

This short and much abbreviated book on Cancer Chemotherapy is directed at practitioners unfamiliar with the management of malignant disease by drug treatment, and at medical students. The clipped precise style of the author may not appeal to every reader, and the condensed style of the book leads to some curious emphases, which I suspect not all oncologists would agree with. If this book is truly intended for the general practitioner who has to deal with patients treated in a specialist hospital, who may develop the complications of chemotherapy at home, it is somewhat surprising that whilst we are informed that busulphan uncommonly causes bronzing of the skin and a wasting syndrome, we are not informed that it may cause cumulative and life-threatening marrow depression in the treatment of chronic myeloid leukaemia.

It is difficult also to place this particular volume in the ever extending library of books by medical oncologists. The format of the book makes it a little indigestible and I feel that it could have been substantially improved with tables and line diagrams.

Most volumes on cancer chemotherapy are out of date by the time of publication because of the rapid changes and this volume is no exception, with some of the references not being updated beyond the early 1970s. The rather negative conclusion of the author also makes the volume difficult to recommend, when she states in her concluding chapter that chemotherapy has little to offer. Certainly, few cures are achieved with chemotherapy at the present time, but the testimony of many patients to the very important palliation that they receive, sometimes over many years, must contradict this particular author's view. This reviewer would personally have valued cautious and rational optimism in conclusion, and I expect that general practitioners looking after patients with malignant disease might find that approach more valuable too.

H. Bush